# Effects of Repetitive-Transcranial Magnetic Stimulation (rTMS) in Fibromyalgia Syndrome: An Umbrella and Mapping Review

**DOI:** 10.3390/brainsci13071059

**Published:** 2023-07-11

**Authors:** Ferran Cuenca-Martínez, Núria Sempere-Rubio, Sara Mollà-Casanova, Elena Muñoz-Gómez, Josué Fernández-Carnero, Alberto Sánchez-Sabater, Luis Suso-Martí

**Affiliations:** 1 Department of Physiotherapy, University of Valencia, 46010 Valencia, Spain; ferran.cuenca@uv.es (F.C.-M.); sara.molla@uv.es (S.M.-C.); elena.munoz-gomez@uv.es (E.M.-G.); alberto.sanchez.sabater@gmail.com (A.S.-S.); luis.suso@uv.es (L.S.-M.); 2 Department of Physical and Occupational Therapy, Rehabilitation and Physical Medicine, Universidad Rey Juan Carlos, 28922 Madrid, Spain; josue.fernandez@urjc.es; 3 La Paz Hospital Institute for Health Research, IdiPAZ, 28922 Madrid, Spain; 4 Grupo de Investigación en Neurociencia Cognitiva, Dolor y Rehabilitación en Ciencias de la Salud (NECODOR), Universidad Rey Juan Carlos, 28922 Madrid, Spain

**Keywords:** transcranial magnetic stimulation, rTMS, fibromyalgia, umbrella review

## Abstract

Background: The main aim of this study was to assess the effects of repetitive-transcranial magnetic stimulation (rTMS) in patients with fibromyalgia (FMS). Methods: We systematically searched PubMed, PEDro, EMBASE, and CINAHL. Methodological quality was analyzed using the AMSTAR and ROBIS scales, and the strength of evidence was established according to the guidelines advisory committee grading criteria. A total of 11 systematic reviews were included. The assessed variables were pain intensity, depressive symptoms, anxiety, and general health. Results: Regarding pain intensity, it seems that high-frequency rTMS significantly reduces pain intensity at a 1-month follow-up when the primary motor cortex (M1) is stimulated. However, we cannot robustly conclude the same for low-frequency protocols. When we look at the combination of high and low-frequency rTMS, there seems to be a significant effect on pain intensity up to 1-week post-intervention, but after that point of follow-up, the results are controversial. Regarding depressive symptoms and anxiety, results showed that the effects of rTMS are almost non-existent. Finally, in regard to general health, results showed that rTMS caused significant post-intervention effects in a robust way. However, the results of the follow-ups are contradictory. Conclusions: The results obtained showed that high-frequency rTMS applied on the M1 showed some effect on the variable of pain intensity with a limited quality of evidence. Overall, rTMS was shown to be effective in improving general health with moderate quality of evidence. Finally, rTMS was not shown to be effective in managing depressive symptoms and anxiety with a limited to moderate quality of evidence. PROSPERO number: This review was previously registered in PROSPERO (CRD42023391032).

## 1. Introduction

Fibromyalgia syndrome (FMS) corresponds to a clinical feature that is mainly characterized by widespread chronic pain that is accompanied by some clinical characteristics such as emotional distress, the presence of fatigue, tender points of widespread pain, and sympathetic nervous system and sleep disturbances [1,2]. Some research studies have recently suggested that one of the mechanisms that may be involved in FMS is a process of central hyperexcitability [3,4]. This process involves the amplification of signaling at the neuronal level in the medullary and supramedullary centers, which may lead to increased sensitivity to pain, lowering the excitability threshold of afferent sensory inputs with painful information [5].

When the central nervous system state of patients with FMS was assessed at the neurophysiological level, alterations in cortical and subcortical processing, with impaired connectivity between the thalamus and premotor areas, the insula, and primary somatosensory areas were found [6]. In addition, an imbalance in GABAergic activity is associated with central sensitization and altered motor excitability of the cortex [7]. When the primary motor cortex (M1) is stimulated, it has been shown that GABA-B receptors are involved in this inhibition [8].

A recent meta-analysis has suggested that patients with fibromyalgia have altered inhibitory–excitatory motor cortex regulation with less cortical inhibition, and this could be modulated by some interventions such as non-invasive brain stimulation, improving the intracortical inhibition, which correlated with less pain [9].

An extensive review conducted by Brighina et al. [10] commented that neuromodulation could be a clinical strategy that might have a relevant role in improving the symptomatology associated with FMS. One of the most important non-invasive brain neuromodulation techniques is repetitive transcranial magnetic stimulation (rTMS). The rTMS technique generates an electromagnetic field on the scalp of the persons subjected to the technique, and by means of this created field, it is able to generate a modulation process in the cortical areas [11]. Depending on the type of application, this modulation may be different. For example, cortical excitability seems to decrease when a low-frequency rTMS protocol is applied, while cortical excitability seems to increase when the protocol is a high-frequency one [12]. In addition, the site of the application seems to be important. Some studies comment that it can be applied to cortical areas related to voluntary movement and pathways related to descending pain inhibition, while it can also be applied to motivational-affective regions [13,14]. However, one of the most studied targets for improving pain has been the M1 [15]. All this is discussed in a study conducted by Knijnik et al. [16]. Currently, the impact of non-invasive neuromodulation techniques in FMS, such as rTMS, is a topic of great interest and has been extensively studied. In fact, several systematic reviews (SRs) with and without meta-analysis (MA) have been published on the subject [15,16,17,18,19,20,21,22,23,24,25]. However, there are contradictory results regarding the effectiveness of rTMS on clinical variables of interest in patients with FMS, such as pain intensity, quality of life, or socio-affective outcomes [15,16,17,18,19,20,21,22,23,24,25]. With the aim of bringing together and critically reviewing all the SRs on rTMS and FMS published to date, the present study was proposed. Therefore, the main aim of this umbrella review was to synthesize the evidence on the effects of rTMS in improving pain intensity, depressive symptoms, anxiety, and general health, compared with sham rTMS intervention in patients with FMS.

## 2. Materials and Methods

This study was conducted in accordance with the preferred reporting items for overviews of systematic reviews, including the harm checklist (PRIO-harms), which consists of 27 items (56 sub-items), followed by a 5-stage process flow diagram (identification, screening, eligibility, inclusion, and separation of relevant studies) [26].

### 2.1. Review Inclusion Criteria

The inclusion criteria employed in this article were based on methodological and clinical factors such as population, intervention, control, outcomes, and study design (PICOS) [27].

#### 2.1.1. Population

The participants selected for the articles were patients with FMS. The included SRs had to explicitly state that they included patients with FMS in their inclusion criteria. Therefore, we excluded all patients with other chronic clinical conditions with persistent pain.

#### 2.1.2. Intervention and Control

We included all SRs comparing the effects of rTMS vs. sham stimulation on patients with FMS. We included rTMS-based interventions when the primary motor cortex (M1) or the dorsolateral prefrontal cortex (DLPFC) was stimulated. In addition, we further divided the results according to whether the stimulation was high (increasing cortical excitability) or low frequency (decreasing cortical excitability). If any research studies included primary studies combining high and low frequency, they were classified as *“combined”*.

#### 2.1.3. Outcome Measures

The measures used to assess the results and effects were variables related to clinical outcomes (pain intensity, depressive symptoms, anxiety, and general health (as a general measure of quality of life)). Table 1 summarizes the instruments used in the primary studies to assess these variables of interest.

#### 2.1.4. Study Design

We selected SRs (with or without MA) of randomized controlled clinical trials (RCCTs) or controlled clinical trials (CCTs) and excluded systematic reviews that included RCCTs or CCTs in combination with non-experimental designs. There were no restrictions for any specific language, as recommended by the international criteria [28].

### 2.2. Search Strategy

We conducted a search for scientific articles published between 1950 and 14 November 2022 in the following databases: PubMed (Medline), PEDro, EMBASE, and CINAHL. The reference sections of the included studies and original studies were screened manually. Appendix A shows the search strategies, which were adapted for each database. The search was conducted by two independent reviewers (FCM and NSR) using the same methodology. Differences that emerged during this phase were resolved by consensus. The reference sections of the original studies were screened manually, and the authors were contacted for further information if necessary.

### 2.3. Selection Criteria and Data Extraction

Initially, two independent reviewers (FCM and NSR) conducted a screening process assessing the relevance of the SR (with and without MA) regarding the studies’ questions and objectives. The first screening process was based on each study’s title information, abstract, and keywords. The full text was reviewed if there was no consensus or if the abstracts contained insufficient information. In the second phase of the screening, the full text was assessed if the studies met all of the inclusion criteria. Data described in the results section were extracted by means of a structured protocol that ensured that the most relevant information was obtained from each study.

### 2.4. Methodological Quality Assessment

The two independent reviewers (FCM and NSR) assessed the methodological quality of the SR (with or without MA), assessing each of the selected studies based on the modified quality assessment scale for systematic reviews (AMSTAR) developed by Barton et al. (2008) [29], a scale shown to be a valid and reliable tool for assessing the methodological quality of systematic reviews. With a total of 13 items, each worth 2 points (with “yes” scoring 2; “in part” scoring 1; “no” scoring 0), the maximum possible score is 26. A high-quality cut-off of 20 or more points was provided by the developers. The exclusion and keyword criteria were modified to better evaluate the selected SRs of this study. In addition, we calculated the kappa coefficient (κ) and percentage (%) agreement scores to assess reliability prior to any consensus and estimated the inter-rater reliability using κ: (1) κ > 0.7 indicates a high level of agreement between the reviewers; (2) κ of 0.5–0.7 indicates a moderate level of agreement; and (3) κ < 0.5 indicates a low level of agreement [30].

### 2.5. Risk of Bias Assessment

We assessed the risk of bias using the risk of bias in SRs tool (ROBIS), which consists of 3 phases: (1) relevance assessment (optional); (2) identification of concerns with the review process through 4 domains related to the study eligibility criteria, identification and selection of studies, data collection and study appraisal and synthesis and findings; and (3) judgment on the risk of bias. The ROBIS tool includes signaling questions to evaluate specific domains to help judge the systematic review’s risk of bias, which should be answered as “yes”, “probably yes”, “probably no”, “no”, or “no information”. The risk of bias is therefore judged as “low”, high”, or “unclear” [31]. The two independent reviewers (FCM and NSR) evaluated the risk of bias in the selected studies. In addition, we calculated the kappa coefficient (κ) and percentage (%) agreement scores to assess reliability prior to any consensus and estimated the inter-rater reliability using κ as described previously.

### 2.6. Grading of Evidence

The physical activity guidelines advisory committee grading criteria (PAGAC) were used to assess the grading of evidence. The criteria used to assess the quality of the evidence were as follows: (1) applicability of the study sample, exposures, and outcomes to the research question, (2) generalizability to the population of interest, (3) risk of bias/study limitations, (4) quantity and consistency of findings across studies, and (5) magnitude and precision of the effect. Using these data, final evidence grades and conclusion statements for each research question were developed [32].

### 2.7. Evidence Map

We created a visual map of the scientific evidence for each SR (with and without MA) to visually display the information. The review information is based on 4 dimensions [33].

a. Nº of studies (bubble size): The size of each bubble is directly proportional to the number of original studies included in each SR.
b. Outcome measures (bubble color): Each assessed variable was determined using a color: (1) pain intensity (blue); (2) depressive symptoms (green); (3) anxiety (orange); and (4) general health (purple).
c. Effect size (x-axis): The authors classified each review according to the effects found. When rTMS showed greater benefits than the sham rTMS, the intervention was classified as “potentially better;” otherwise, the intervention was classified as “potentially worse”, If there were no differences, the intervention was included as “no differences”. If there were contradictory results, we included the intervention as “mixed results”.
d. Strength of findings (y-axis): AMSTAR

## 3. Results

### 3.1. Study Selection

**Figure 1:**
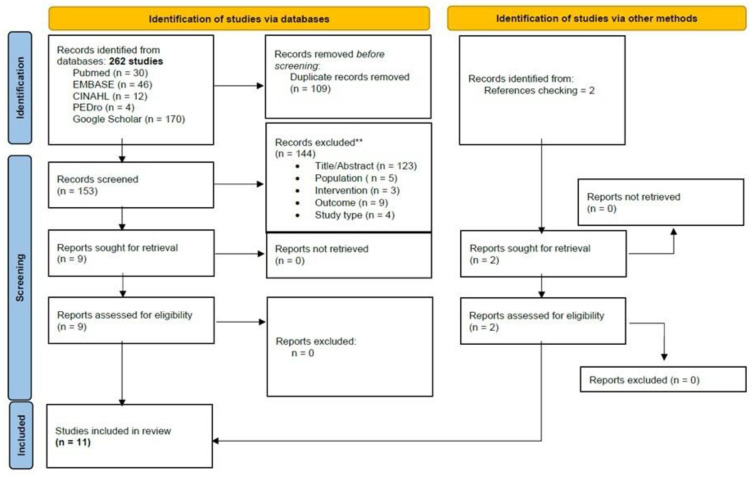
PRISMA Flowchart of studies selection.

The initial search revealed 262 records. An additional two were retrieved manually from the references. Through the title and abstract screening and the full-text assessment, 11 SRs were eligible according to our criteria. The study screening strategy is shown in the form of a flow chart (Figure 1).

### 3.2. Characteristics of the Included Systematic Reviews

Table 1 lists the characteristics of the systematic reviews included (study design, original studies included, demographic characteristics, interventions, variables, and results). Table 1 shows that most of the studies report the diagnostic criteria for patients with FMS and, moreover, that they all offer a wealth of information regarding the parameters for the application of rTMS techniques, as well as the instruments used to evaluate the main outcome measures.

### 3.3. Results of the Methodological Quality (AMSTAR)

The scores ranged from 13 to 24 points out of a possible 26, with a mean score of 20.18 ± 2.96 points. Six studies [15,16,18,20,21,24] (55.5%) scored above 20 points and were considered high-quality (Table 2). The items with the highest scores were those related to “explicitly described to allow replication”, “adequate number and range of databases”, and “quality assessment explicitly described to allow replication”. The lowest scoring item was “non-English-language papers included in the search” and “conclusions address level of evidence for each intervention/comparison”. The inter-rater reliability of the methodological quality assessment was high (κ = 0.831).

### 3.4. Results of Risk of Bias

**Figure 2:**
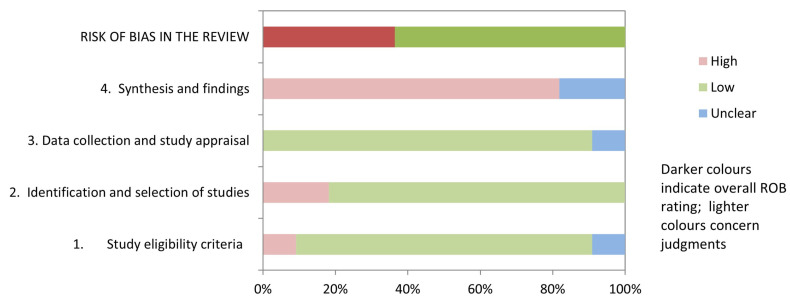
Graphical representation for ROBIS results.

Table 3 and Figure 2 show the results of the risk of bias assessment using ROBIS. 36.3% of studies (4/11) had a low risk of bias. The domains related to the “study eligibility criteria” and the “data collection and study appraisal” had the lowest risk of bias (86.3%). In contrast, the domain related to the “synthesis and findings” had the highest risk of bias (81.8%). The inter-rater reliability for the risk of bias assessment was high (κ = 0.797).

### 3.5. Grading of Evidence Results (PAGAC)

Table 4 shows the findings regarding the quality of evidence for each outcome of the research question. The quality of evidence found for pain intensity and anxiety was limited. Finally, the quality of evidence found for depressive symptoms and general health was moderate.

### 3.6. Evidence Map

**Figure 3:**
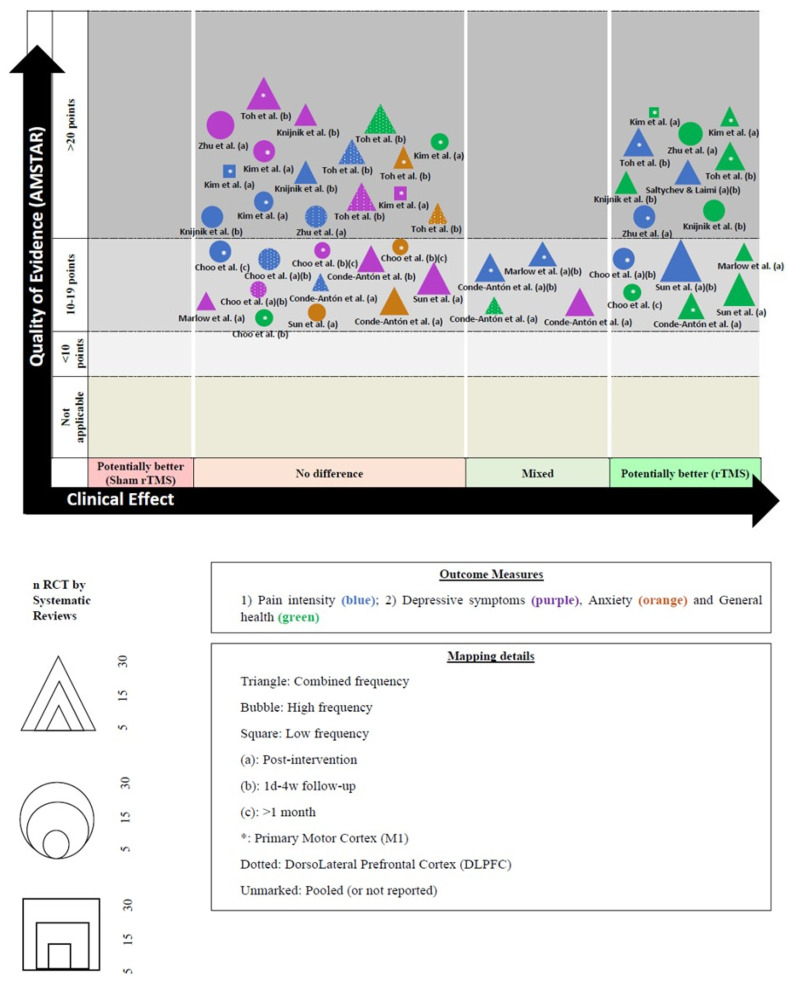
Evidence map and figure legend. * abbreviations [16,17,18,19,20,21,22,24,25].

Figure 3 shows the results pooled in an evidence map.

### 3.7. Outcome Measures

#### 3.7.1. Pain Intensity

A total of 11 studies assessed the pain intensity variable [15,16,17,18,19,20,21,22,23,24,25]. Su et al. [23] were the only ones who conducted analyses using instruments as well as variables. Thus, they reported three outcomes for the pain intensity variables (FMS-related pain intensity (visual analog scale/numeric pain rating scale), interference subscale of brief pain inventory (BPI), and McGill pain questionnaire (MPQ)).

##### High-Frequency rTMS

Five studies assessed the effects of high-frequency rTMS on pain intensity variables [16,17,20,23,24]. In general, contradictory results were found. For example, Choo et al. [17] found a significant effect on pain reduction immediately (number of studies analyzed (*n*) = 4, standardized mean difference (SMD) = 1.23, 95% CI 0.14 to 2.31, *p* = 0.03, inconsistency index (I^2^) = 87%) and 1–4 weeks after the end of the session (*n* = 4, SMD = 0.43, 95% CI 0.10 to 0.77, *p* = 0.01, I^2^ = 42%) but no significant effect after 5–12 weeks (*n* = 2, SMD = 0.14, 95% CI −0.35 to 0.63, *p* = 0.57, I^2^ = 0%) when rTMS was applied to the M1. In contrast, rTMS on the left DLPFC did not reduce pain from fibromyalgia (*n* = 4, SMD = −0.47, 95% CI −1.88 to 0.93, *p* = 0.51, I^2^ = 85% immediately, and *n* = 4, SMD = 0.71, 95% CI −0.07 to 1.50, *p* = 0.08, I^2^ = 58% at 1–4 weeks after the end of treatment) [17]. Knijnik et al. [16] conducted a sensitivity analysis with the aim of excluding the only study arm using low-frequency rTMS. Therefore, Knijnik et al. [16] reported no significant effects on pain reduction using high-frequency rTMS 4 weeks after the end of the intervention (*n* = 4, SMD = −0.293, 95% CI −0.63 to 0.04, *p* = 0.09, I^2^ = 35.7%). Kim et al. [20] conducted a sensitivity analysis, and they found no significant effect on pain reduction when high-frequency rTMS was applied for at least 10 sessions to the M1 region (*n* = 3, mean differences (MD) = −0.86, 95% CI −2.59 to 0.87, *p* > 0.05, I^2^ = 89%). Su et al. [23] conducted various subgroup analyses. They found a significant effect on the interference subscale of BPI immediately after a high-frequency rTMS intervention (SMD = −0.48, 95% CI −0.91 to −0.05, *p* < 0.05) and between 2 weeks and 1 month of follow-up (SMD = −0.56, 95% CI −0.96 to −0.16, *p* < 0.05). In addition, Su et al. [23] found a significant effect on FMS-related pain intensity immediately after a high-frequency rTMS intervention (SMD = −0.80, 95% CI −1.16 to −0.45, *p* < 0.05), between 2 weeks and 1 month of follow-up (SMD = −0.48, 95% CI −0.80 to −0.16, *p* < 0.05) but not between 1.5 and 3 months of follow-up (SMD = −0.49, 95% CI −1.1 to 0.11, *p* > 0.05). Finally, Su et al. [23] showed no significant effect on the MPQ immediately after a high-frequency rTMS intervention (SMD = −0.48, 95% CI −0.98 to 0.01, *p* > 0.05). However, they found significant differences between 2 weeks and 1 month of follow-up (SMD = −0.66, 95% CI −1.0 to −0.25, *p* < 0.05). Zhu et al. [24] found a significant effect on pain reduction after a high-frequency rTMS at 10 Hz intervention to the M1 (*n* = 4, SMD = −0.98, 95% CI −1.54 to −0.43, *p* < 0.001, I^2^ = 54%) but not when rTMS at 10 Hz was applied to the DLPFC (*n* = 4, SMD = −0.38, 95% CI −0.83 to 0.06, *p* > 0.05, I^2^ = 46%). In conclusion, three of the four studies (only one assessed effect after one month of follow-up) found a significant reduction in post-intervention pain intensity when the M1 was stimulated, and this improvement seems to have been sustained between one and four weeks but never longer than four weeks post-intervention. What does appear to be robust is that pain intensity never improved when the DLPFC cortex was stimulated.

##### Low-Frequency

Two studies assessed the effects of low-frequency rTMS on pain intensity variables [20,23]. In general, contradictory results were also found. Kim et al. [20] also conducted a sensitivity analysis, and they found no significant effect on pain reduction when low-frequency rTMS was applied for at least 10 sessions to the M1 region (*n* = 1, MD = −1.31, 95% CI −2.65 to 0.03, *p* > 0.05). Su et al. [23] conducted various subgroup analyses. They found a significant effect on FMS-related pain intensity immediately after a low-frequency rTMS intervention (SMD = −0.65, 95% CI −0.98 to −0.31, *p* < 0.05), between 2 weeks and 1 month of follow-up (SMD = −0.55, 95% CI −0.88 to −0.21, *p* < 0.05) and also between 1.5 and 3 months of follow-up (SMD = −0.61, 95% CI −1.0 to −0.21, *p* < 0.05). Finally, Su et al. [23] showed a significant effect on the MPQ immediately after a low-frequency rTMS intervention (SMD = −0.73, 95% CI −1.1 to −0.29, *p* < 0.05) and between 2 weeks to 1 month of follow-up (SMD = −0.73, 95% CI −1.1 to −0.29, *p* < 0.05). In conclusion, it is difficult to make an ending for the effect of low frequency on pain intensity because the sensitivity analysis of Kim et al. [20] is only based on one primary study, and Su et al. [23] does not fully clarify the stimulation zones.

##### Combined-Frequency

Seven studies assessed the effects of combined (high and low) frequency rTMS on the pain intensity variable [15,16,18,19,21,22,25]. Knijnik et al. [16] found no significant effect on pain reduction 30 days after rTMS intervention (*n* = 5, SMD = −0.31, 95% CI −0.64 to 0.02, *p* = 0.063, I^2^ = 22%). Hou et al. [15] showed a significant effect on pain reduction between 1 day and 25 weeks after the intervention when rTMS was applied to the M1 (SMD = 0.67, 95% CI 0.124 to 1.216, *p* < 0.001) and also when rTMS was applied to the DLPFC (SMD = 0.708, 95% CI 0.24 to 1.175, *p* < 0.001). Saltychev & Laimi [18] found a significant effect immediately also between 1 week and 1 month post-intervention on pain reduction (*n* = 6, MD = −1.2, 95% CI −1.7 to −0.8, *p* < 0.05, I^2^ = 42% and, *n* = 6, MD = −0.7, 95% CI −1.0 to −0.3, *p* < 0.05, I^2^ = 0%, respectively). Conde-Antón et al. [19] conducted a systematic review without statistical aggregation, and they found controversial results on pain reduction in the short and medium-term when rTMS intervention was applied to the M1 (significant and non-significant results, *n* = 7). However, Conde-Antón et al. [19] found no significant results on pain reduction when rTMS intervention was applied to the DLPFC (*n* = 2). Toh et al. [21] conducted a sensitivity analysis, and they found a significant effect on pain reduction 4 weeks after rTMS intervention on the M1 (*n* = 7, SMD = −0.57, 95% CI −0.91 to 0.23, *p* < 0.05, I^2^ = 6%) but not when rTMS was applied to the DLPFC (*n* = 6, SMD = −0.31, 95% CI −0.70 to 0.08, *p* > 0.05, I^2^ = 0%). Sun et al. [22] found a significant effect on pain reduction either immediately after treatment or after 1 week (*n* = 14, SMD = −0.49, 95% CI −0.86 to −0.13, *p* = 0.008, I^2^ = 68%). Marlow et al. [25] conducted an SR without statistical aggregation, and they found controversial results on pain reduction in the short and medium term (significant and non-significant results, *n* = 5). In conclusion, two SRs without MA show inconsistency, with findings in favor and against the technique (when evaluated together regardless of frequency). If we look at SRs with MA, we find that pain intensity decreases significantly immediately after the end of the intervention and also 1 week post-intervention, but the results are controversial after 1 month of follow-up (where we find significant and non-significant effects).

#### 3.7.2. Depressive Symptoms

A total of 10 studies assessed the depressive symptoms variable [15,16,17,19,20,21,22,23,24,25]. Su et al. [23] were the only ones who conducted analyses using instruments as well as variables. Thus, they reported two outcomes for the depressive symptoms variable (Beck Depression Inventory (BDI) score and Hamilton Depression Rating Scale (HDRS)).

##### High-Frequency

Four studies assessed the effects of high-frequency rTMS on depressive symptoms variable [17,20,23,24]. Choo et al. [17] found no significant effect after rTMS treatment on either the left M1 (*n* = 2, SMD = 0.11, 95% CI −0.33 to 0.56, *p* = 0.62, I^2^ = 0% at 1–4 weeks after the end of treatment, and *n* = 2, SMD = 0.19, 95% CI −0.25 to0.64, *p* = 0.40, I^2^ = 30% at 5–12 weeks after the end of rTMS) or the left DLPFC (*n* = 2, SMD = 0.71, 95% CI −1.07–2.49, *p* = 0.43, I^2^ = 84% immediately, and *n* = 2, SMD = 0.56, 95% CI −0.77 to 1.90, *p* = 0.41, I^2^ = 77% at 1–4 weeks after the end of treatment). Kim et al. [20] found no significant effect on depressive symptoms after rTMS intervention on the M1 (*n* = 4, MD = −0.37, 95% CI −2.94 to 2.19, *p* > 0.05, I^2^ = 81%). Su et al. [23] conducted various subgroup analyses. They found a significant effect on BDI immediately after a high-frequency rTMS intervention (SMD = −0.38, 95% CI −0.69 to −0.08, *p* < 0.05) and between 2 weeks and 1 month of follow-up (SMD = −0.41, 95% CI −0.76 to −0.07, *p* < 0.05. However, Su et al. [23] found no significant effect on HDRS immediately after high-frequency rTMS intervention (SMD = −0.38, 95% CI −0.88 to 0.11, *p* > 0.05), between 2 weeks and 1 month of follow-up (SMD = −0.4, 95% CI −0.9 to 0.09, *p* > 0.05) nor between 1.5 and 3 months of follow-up (SMD = −0.35, 95% CI −0.95 to 0.24, *p* > 0.05). Zhu et al. [24] found no significant effect on depressive symptoms after a high-frequency rTMS at 10 Hz intervention (*n* = 7, SMD = −0.23, 95% CI −0.5 to 0.05, *p* = 0.11, I^2^ = 33%). Overall, the results seem fairly consistent regarding the impact of high-frequency rTMS on depressive symptoms. Three studies find no significant effect either at the end of the intervention or at any follow-up point. There is also no difference between stimulating the M1 and the DLPFC. Only one study shows contradictory results [23]. Upon evaluating the effect of rTMS intervention on the BDI questionnaire, significant results are found up to 1 month of follow-up, although with a low effect size; however, when the HDRS questionnaire was used, the results were not statistically significant at any point in the evaluation. It seems that the effect of high-frequency rTMS on depressive symptoms is almost non-existent.

##### Low-Frequency

Two studies assessed the effects of low-frequency rTMS on depressive symptoms variable [20,23]. Kim et al. [20] found no significant effect on depressive symptoms after rTMS intervention on the M1 (*n* = 1, MD = 4.33, 95% CI −1.42 to 10.08, *p* > 0.05). Su et al. [23] found no significant effect on BDI immediately after a low-frequency rTMS intervention (SMD = −0.39, 95% CI −1.1 to 0.3, *p* > 0.05) nor in between 2 weeks and 1 month of follow-up (SMD = −0.19, 95% CI −0.89 to 0.5, *p* > 0.05). In addition, Su et al. [23] found no significant effect on HDRS immediately after low-frequency rTMS intervention (SMD = −0.43, 95% CI −1.1 to 0.26, *p* > 0.05). However, they found a significant effect on HDRS between 2 weeks and 1 month of follow-up (SMD = −0.61, 95% CI −1.0 to −0.21, *p* < 0.05) and between 1.5 and 3 months of follow-up (SMD = −0.73, 95% CI −1.1 to −0.29, *p* < 0.05). In conclusion, the results showed that low-frequency rTMS stimulation did not elicit significant post-intervention effects on depressive symptoms. However, contradictory results were found at follow-up, with significant and non-significant effects. However, we must take these results with caution because they derive from a single SR that differentiates results according to the questionnaire used [23]. 

##### Combined-Frequency

Six studies assessed the effects of combined (high and low) frequency rTMS on depressive symptoms variable [15,16,19,21,22,25]. Knijnik et al. [16] showed no significant effect on depressive symptoms 30 days after rTMS intervention (*n* = 3, SMD = −0.15, 95% CI −0.47 to 0.17, *p* = 0.36, I^2^ = 0%). Hou et al. [15] showed a significant effect on depressive symptoms between 1 day and 25 weeks after the intervention when rTMS was applied only to the DLPFC (SMD = 0.543, 95% CI 0.074 to 0.993, *p* < 0.001), but not when rTMS was applied to the M1 (SMD = 0.246, 95% CI −0.056 to 0.549, *p* > 0.05). Conde-Antón et al. [19] showed controversial results regarding the improvement of depressive symptoms in the short term (significant and non-significant results, *n* = 7). However, they found no differences in the medium term (*n* = 6). Toh et al. [21] found no significant effect on depressive symptoms 4 weeks after rTMS intervention to the M1 (*n* = 7, SMD = −0.17, 95% CI −0.50 to 0.16, *p* > 0.05, I^2^ = 22%) nor the DLPFC (*n* = 6, SMD = −0.10, 95% CI −0.49 to 0.29, *p* > 0.05, I^2^ = 0%). Sun et al. [22] found no significant effect on depressive symptoms after rTMS intervention (*n* = 9, SMD = −0.13, 95% CI −0.39 to 0.13, *p* = 0.33, I^2^ = 27%). Marlow et al. [25] found no significant results on depressive symptoms in the short term (*n* = 2). Overall, the results were contradictory but led to little or no effect. Four studies found no effect of rTMS on depressive symptoms either at post-intervention or one-month follow-up. One study found significant and non-significant results at post-intervention but not at follow-up. Only one study found significant differences when the DLPFC was stimulated but not when the M1 was stimulated.

#### 3.7.3. Anxiety

A total of six studies assessed the anxiety variable [15,17,19,21,22,23].

##### High-Frequency

Three studies assessed the effects of high-frequency rTMS on the anxiety variable [17,22,23]. Choo et al. [17] found no significant effect on anxiety 1–4 weeks after the end of rTMS intervention on the left M1 (*n* = 2, SMD = −0.38, 95% CI −0.83 to 0.07, *p* = 0.10, I^2^ = 0%), and also at 5–12 weeks after the end of rTMS (*n* = 2, SMD = 0.01, 95% CI −0.44 to 0.45, *p* = 0.97, I^2^ = 0%). Sun et al. [22] found no significant effect on anxiety after rTMS intervention (*n* = 3, SMD = 0.10, 95% CI −0.34 to 0.53, *p* = 0.67, I^2^ = 0%). Su et al. [23] found a significant effect on anxiety after rTMS intervention (*n* = 3, SMD = −0.6, 95% CI −1.0 to −0.13, *p* < 0.05, I^2^ = 8.9%). Overall, there are conflicting results on the post-intervention effect of high-frequency rTMS on anxiety (one study found evidence in favor and one did not), and no effect was found at any follow-up point.

##### Combined-Frequency

Three studies assessed the effects of combined (high and low) frequency rTMS on the anxiety variable [15,19,21]. Hou et al. [15] found no significant effect on anxiety between 1 day and 25 weeks after the intervention when rTMS was applied to the M1 (SMD = 0.162, 95% CI −0.243 to 0.568, *p* > 0.05). Conde-Antón et al. [19] showed no significant effect on anxiety after rTMS intervention (*n* = 5). Toh et al. [21] found no significant effect on anxiety 4 weeks after rTMS intervention to the M1 (*n* = 3, SMD = 0.0, 95% CI −0.39 to 0.40, *p* > 0.05, I^2^ = 0%) nor to the DLPFC (*n* = 2, SMD = 0.15, 95% CI −0.43 to 0.73, *p* > 0.05, I^2^ = 0%). The result was robust. No significant effect in favor of rTMS (combined frequency) on anxiety was found either at post-intervention or at any follow-up point.

#### 3.7.4. General Health

A total of 10 studies assessed the general health variable [15,16,17,19,20,21,22,23,24,25].

##### High-Frequency

Four studies assessed the effects of high-frequency rTMS on the general health variable [16,17,23,24]. Choo et al. [17] found a significant effect on the general health variable after 5–12 weeks on the left M1 (*n* = 2, SMD = 0.93, 95% CI 0.46 to 1.40, *p* < 0.01, I^2^ = 0%) but had no significant effect after 1–4 weeks on the left M1 (*n* = 2, SMD = 0.67, 95% CI −0.52 to 1.86, *p* = 0.27, I^2^ = 85%). Knijnik et al. [16] conducted a sensitivity analysis with the aim of excluding the only study arm using low-frequency rTMS, and they found a significant effect on the general health variable 4 weeks after rTMS intervention (*n* = 4, SMD = −0.46, 95% CI −0.80 to −0.11, *p* = 0.01, I^2^ = 0%). Su et al. [23] found a significant effect on general health after high-frequency rTMS intervention (SMD = −0.66, 95% CI −1.22 to −0.1, *p* < 0.05) and between 2 weeks and 1 month of follow-up (SMD = −0.82, 95% CI −1.27 to −0.37, *p* < 0.05). Zhu et al. [24] found a significant effect on general health after high-frequency rTMS intervention at 10 Hz (*n* = 6, SMD = −0.70, 95% CI −1.0 to −0.4, *p* < 0.001, I^2^ = 15%). In conclusion, it seems that the results showed a significant immediate post-intervention effect of high-frequency rTMS on general health in all studies. The results are contradictory between 1 week and 1 month of follow-up, but after 1 month, we found significant results in favor of high-frequency rTMS for up to 12 weeks post-intervention.

##### Low-Frequency

Only one study assessed the effect of low-frequency rTMS on general health [23]. Su et al. [23] found a significant effect on general health after low-frequency rTMS intervention (SMD = −0.9, 95% CI −1.63 to −0.17, *p* < 0.05) but not between 2 weeks and 1 month of follow-up (SMD = −0.67, 95% CI −1.38 to 0.04, *p* < 0.05). It appears, therefore, that low-frequency rTMS has a post-intervention impact on improving general health but not beyond that assessment point.

##### Combined-Frequency

Seven studies assessed the effects of combined (high and low) frequency rTMS on the general health variable [15,16,19,20,21,22,25]. Knijnik et al. [16] showed a significant effect on general health 4 weeks after rTMS intervention (*n* = 5, SMD = −0.47, 95% CI −0.80 to −0.11, *p* = 0.01, I^2^ = 0%). Hou et al. [15] showed a significant effect on general health between 1 day and 25 weeks after the intervention when rTMS was applied to the M1 (SMD = 0.581, 95% CI 0.219 to 0.943, *p* < 0.001) and when rTMS was applied to the DLPFC (SMD = 0.631, 95% CI 0.065 to 1.197, *p* < 0.001). Conde-Antón et al. [19] found a significant effect on the improvement of some subdomains of general health in the short term when rTMS intervention was applied to the M1 (*n* = 7). However, when applied to the DLPFC, contradictory results were found (*n* = 2). Kim et al. [20] found a significant effect on the improvement of general health when rTMS intervention was applied to the M1 (*n* = 3, MD = −2.5, 95% CI −3.99 to −1.01, I^2^ = 0%, *p* < 0.05). However, when the impact on FMS was assessed, controversial results were found. Kim et al. [20] found statistically significant differences when low-frequency rTMS was applied to the M1 (*n* = 1, MD = 15.02, 95% CI 5.59 to 24.45, *p* < 0.05) but not when high-frequency rTMS was applied (*n* = 3, MD = 2.8, 95% CI −5.51 to 11.11, *p* > 0.05, I^2^ = 72%). Toh et al. [21] showed a significant effect on general health 4 weeks after rTMS intervention on the M1 (*n* = 6, SMD = −0.82, 95% CI −1.16 to −0.47, *p* < 0.05, I^2^ = 0%) but not when rTMS was applied to the DLPFC (*n* = 6, SMD = −0.13, 95% CI −0.52 to 0.26, *p* > 0.05, I^2^ = 0%). Sun et al. [22] found a significant effect on general health after rTMS treatment (*n* = 10, SMD = −0.50, 95% CI −0.75 to −0.25, *p* < 0.001, I^2^ = 28%). Finally, Marlow et al. [25] found significant improvements in general health in the short term (*n* = 3). In conclusion, the results were generally consistent regarding the effect of rTMS (when analyzed as high and low-frequency combined interventions) on general health in patients with FMS. The findings show that rTMS had a significant effect both immediately after the end of the intervention and at four weeks post-intervention, and one study even showed significant differences at twenty five weeks of follow-up. Finally, it should be noted that there seems to be greater effectiveness if the M1 is stimulated rather than the DLPFC.

## 4. Discussion

### 4.1. Principal Results

The main aim of this umbrella and mapping review was to synthesize evidence on the effects of rTMS in improving pain intensity, depressive symptoms, anxiety, and general health, compared with sham rTMS interventions in patients with FMS. Regarding pain intensity, we can group and highlight some interesting results. It seems that high-frequency rTMS protocols have a significant effect on reducing pain intensity for up to 1 month of follow-up when the M1 but not the DLPFC is stimulated. In addition to this, we cannot robustly conclude the same for low-frequency protocols. Finally, when we look at the combination of high and low-frequency rTMS, there seems to be a significant effect on pain intensity up to 1-week post-intervention, but after that point of follow-up, the results are controversial. These results are consistent with the current literature, which suggests that the analgesic effects of M1 and DLPFC stimulation may act upon different mechanisms [34]. It seems that pain inhibition phenomena may be more pronounced when the M1 is stimulated. However, little is known about the neurobiological basis for this unconventional role of the M1 in modulating pain perception. Pain perception evokes responses in the human M1 in intracortical recordings and functional neuroimaging studies [35]. It has been suggested that M1 plasticity underlies chronic pain [36], and the analgesic effects of M1 stimulation can change thalamic and subthalamic nuclei and modulate the affective components of pain [37]. In addition, M1 stimulation could evoke motor disinhibition that could decrease chronic pain [38]. In this sense, Gan et al. [39] recently reviewed the neurophysiological pathways related to hypoalgesia following M1 stimulation. These authors found a connection between the M1 and the nucleus accumbens, stimulating a reward circuit that could inhibit negative emotional responses related to neuropathic pain. In addition, they also found connections with the periaqueductal gray matter, which could be related to a suppression of sensory sensitivity [39].

With respect to depressive symptoms, it seems that the effects of high and low-frequency rTMS are almost non-existent in the improvement of these symptoms. Finally, when we analyzed both forms of stimulation in combination, the results were contradictory but led to little or no effect. Pooling the findings, it appears that rTMS, regardless of the type of stimulation frequency, seems to be ineffective in improving depressive symptoms in patients with FMS. In relation to anxiety, we found similar results as with depressive symptoms. Contradictory post-intervention results were found when analyzing high-frequency rTMS, but no effect was found beyond that assessment point. When we analyze both frequencies combined, there is no significant effect on anxiety either immediately after the end of the intervention or at any follow-up point. The effect, therefore, of rTMS on anxiety in FMS patients seems almost non-existent.

Finally, if we look at general health, it seems that high and low-frequency rTMS showed significant post-intervention effects in a robust way. However, the results of follow-ups are contradictory. Finally, when we analyzed the protocols in combination, rTMS elicited a significant effect at the end of the intervention and 1-month post-intervention. Importantly, M1 stimulation elicited a greater effect than when the DLPFC was stimulated. The neurophysiological bases explaining the results of M1 high-frequency rTMS stimulation in depressive symptoms or anxiety are various. The limbic system is related to many other cortical regions, such as the temporal lobe, which is related to social cognition [40]. Therefore, the limbic system and right medial temporal cortex are involved in the control of pain-related emotional aspects in emotion modulation [41]. Therefore, the neural connections caused by stimulation of the M1 may affect affective variables by activating social cognition and emotional modulation, as well as changing resting-state functional connectivity in affective processing areas [42].

### 4.2. Study Limitations

This study has a number of limitations that should be taken into consideration. First, we have had to provide a result that groups the effects of high and low-frequency protocols (at the same time) because the SRs and MA have grouped them in this way. This really makes little sense because one protocol aims to increase the excitability of the cortex while the other aims to decrease it. Grouping the effects by protocol (probably due to low primary studies) undermines the justification of the technique, and it would make more sense to group all the high-frequency effects on the one hand and all the low-frequency effects on the other. This should be taken into consideration. Finally, we were unable to find the number of articles included in the subgroup analyses conducted by Hou et al. [15] and Su et al. [23], so the mapping lost some relevant information.

### 4.3. Strong Points of This Review

The main strength of this research review is that it systematically combines everything published to date regarding non-invasive brain stimulation, through rTMS, in patients with FMS. This research design allows us to critically and systematically compile all the systematic review studies, with or without statistical aggregation, to evaluate critical aspects at the scientific level and the clinical level, such as the brain stimulation parameters used, the number of sessions, type of patients, assessment instruments used, etc. Future studies should take into account some of the considerations reported in this research to obtain more robust results.

## 5. Conclusions

The results showed that high-frequency rTMS protocols, applied over the M1, seem to have a significant effect on reducing pain intensity up to at least 1 month of follow-up but never when applied over the DLPFC. In addition, rTMS appears to be ineffective regardless of protocol and area of application on depressive and anxiety symptoms. Finally, regarding general health, the results showed that high and low-frequency rTMS protocols resulted in significant post-intervention improvements. This latter result was the most consensual result in the assessed SRs.

## Figures and Tables

**Table 1:** Characteristics of the reviews included in the umbrella review.

Study	Studies k (*n*) Types	Meta-Analysis (k)	Population	Intervention	Control	Outcomes	Author’s Conclusions
Choo et al. [17]	10 (299) RCTs	Yes (10)	FMS (Diagnosis criteria were not specified)	High-frequency rTMS: 10 Hz Stimulation areas: M1 DLPFC	Sham stimulation	-Pain intensity (VAS, BIRS, BPI, NRS, SF-MPQ, MPQ) -Anxiety and depressive symptoms (HADS, MADRS, BDI, HDRS) -General health (FIQ, K-FIQ, SF36, WHQOL-BREF)	High-frequency rTMS had a positive pain-reducing effect immediately and at 1–4 weeks after completion of rTMS sessions, and the patient’s general health improved after 5–12 weeks. However, DLPFC stimulation was not effective in controlling fibromyalgia symptoms.
Knijnik et al. [16]	5 (143) RCTs	Yes (5)	FMS (Diagnosis based on 1990 ACR criteria)	High-frequency rTMS: 10 Hz One study used low-frequency rTMS: 1 Hz Stimulation areas: Left M1 Left DLPFC Right DLPFC	Sham stimulation	-Pain intensity (BPI, VAS, average pain intensity over the last 24 h) -Depressive symptoms (BDI, HDRS) -General health (FIQ)	In comparison with sham stimulation, rTMS demonstrated a superior effect on the general health of patients with FMS 1 month after starting therapy. These statistically significant changes were not found in depression and pain intensity.
Hou et al. [15]	11 (rTMS) (369) RCTs	Yes (16)	FMS (Two versions of diagnostic criteria: ACR 1990 and 2010)	High-frequency rTMS (10 Hz) Low-frequency rTMS (1 Hz) + medication and/or psychotherapy Stimulation areas: M1 DLPFC Total stimulation doses varied from 12,000 to 45,000 pulses in rTMS. The intervals of treatment ranged from 5 days to 22 weeks.	Sham stimulation + medication and/or psychotherapy	-Pain intensity (VAS, NRS, SF-MPQ, SF-BPI, BURS, BIRS) -Depressive symptoms (BDI, HDRS, MADRS, HADS) -General health (SF-36, FIQ, K-FIQ, WHOQOL-BREF, MOS-SF-12, CGIS)	M1 stimulation may be better in pain reduction, and the dorsolateral prefrontal cortex may be better in depression improvement in patients with FMS.
Saltychev & Laimi [18]	8 (294) RCTs	Yes (6)	FMS (Diagnosis criteria were not specified)	High-frequency rTMS (10 Hz) Low-intensity TMS (1 Hz) Stimulation areas: Left M1 Right DLPFC Left DLPFC 2 studies used low-intensity rTMS: Right DLPFC Diffuse application	Sham stimulation	Pain intensity (NRS)	There is moderate evidence that rTMS is not more effective than sham in reducing the severity of pain in FMS.
Conde-Antón et al. [19]	8 (rTMS) (308) RCTs	No	FMS (Diagnosis based on 1990 ACR criteria)	High-frequency rTMS (10 Hz) Low-intensity TMS (1 Hz) Stimulation areas: M1 DLPFC The pulses per session ranged from 1200 to 2000.	Sham stimulation	-Pain intensity (VAS, BPI, NRS, SF-MPQ) -Anxiety and depressive symptoms (HADS, MADRS, BDI, BAI, HDRS) -General health (FIQ, SF36, WHQOL-BREF)	Results showed significant effects on pain intensity and general health but not on depressive and anxiety symptoms in patients with FMS.
Kim et al. [20]	5 (85) RCTs	Yes (5)	FMS (Diagnosis criteria were not specified)	High-frequency rTMS: 10 Hz In the selected studies, rTMS was applied at least 10 sessions to the M1. The pulses per session ranged from 1200 to 1500.	Sham stimulation	-Pain intensity (VAS, NPRS) -Depressive symptoms (BDI, MADRS) -General health (SF-36, WHOQOL, FIQ)	Results showed statistically significant results in general health but not in pain intensity and depressive symptoms in patients with FMS.
Toh et al. [21]	11 (303) RCTs	Yes (11)	FMS (Diagnosis criteria were not specified)	High-frequency rTMS (10 Hz) Stimulation areas: Five studies applied rTMS to the M1 alone. Four studies applied rTMS to the DLPFC alone. Two studies applied rTMS to both the M1 and DLPFC separately.	Sham stimulation.	-Pain intensity (VAS, NRS, BPI, MPQ) -Anxiety and depressive symptoms (BDI, HDRS, HADS, BAI, MADRS) -General health (FIQ, SF-36)	rTMS is more effective than sham in improving pain and quality of life, but it does not demonstrate a reduction in depression or anxiety in patients with FMS.
Sun et al. [22]	14 (433) RCTs	Yes (12)	FMS (Diagnosis criteria were not specified)	High-frequency (10 Hz) and low-frequency (1 Hz) Stimulation areas: M1 DLPFC The total amount of pulses in a session, multiplied by the number of sessions, varies from 12,000 to 60,000 pulses of total stimulation, mostly 12,000–40,000.	Sham stimulation	-Pain intensity (NPRS, VAS, BPI, MPQ, SF-MPQ, BURS, BIRS) -Anxiety and depressive symptoms (BDI, BAI HADS, HDRS, MADRS) -General health (SF-36, FIQ, WHOQOL-BREF, CGIS)	Results showed that rTMS relieved pain and enhanced the general health of patients with FMS; however, on the basis of current reports, it did not improve anxiety and depression.
Su et al. [23]	18 (643) RCTs	Yes (17)	FMS (Four versions of diagnostic criteria were included: ACR 1990, 2010, 2011, and 2016)	High-frequency (10 Hz) and low-frequency (1 Hz) + medication and/or multicomponent therapy program Stimulation areas: M1 DLPFC The total number of pulses ranged from 12,000 to 60,000, and the pulses per session ranged from 1200 to 4000.	Sham stimulation + medication and/or multicomponent therapy program	-Pain intensity (NPRS, VAS, BPI, MPQ, SF-MPQ, BURS, BIRS) -Anxiety and depressive symptoms (BDI, BAI HADS, HDRS, MADRS) -General health (SF-36, FIQ, WHOQOL-BREF, CGIS)	Reductions in general health, pain, depression, and anxiety were discovered, which persisted for at least two weeks after the last intervention in patients with FMS.
Zhu et al. [24]	7 (217) RCTs	Yes (7)	FMS (Diagnosis based on ACR criteria)	High-frequency (10 Hz) Stimulation areas: M1 DLPFC	Sham stimulation	-Pain intensity (VAS, BPI) -Depressive symptoms (BDI, HDRS) -General health (FIQ)	Overall, 10-Hz rTMS had a significant effect on analgesia and improved general health in patients with FMS but did not improve depression.
Marlow et al. [25]	5 (120) RCTs and case series (1 study)	No	FMS (Diagnosis based on 1990 ACR criteria)	High-frequency (10 Hz) and low-frequency (1 Hz) Stimulation areas: M1 DLPFC The pulses per session ranged from 1200 to 2000.	Sham stimulation	-Pain intensity (VAS, BPI, NRS, MPQ) -Depressive symptoms (BDI, HDRS, MADRS, HADS) -General health (FIQ, CGI, GAF)	Results showed controversial findings with regard to pain intensity. In addition, the study showed significant changes in general health but not depressive symptoms.

Notes: ACR: American College of Rheumatology; RCT: Randomized Controlled Trial; FMS: Fibromyalgia syndrome; DLPFC: Dorsolateral Prefrontal Cortex; M1: Primary Motor Cortex; SF-36: 36-Item Short-Form Health Survey; HDRS: Hamilton Depression Rating Scale; BIRS: Gracely Box Intensity Scale; BPI: Brief Pain Inventory; SF-BPI: Short Form BPI; MPQ: McGill Pain Questionnaire; SF-MPQ: Short Form McGill Pain Questionnaire NRS: numeric rating scale; MADRS: Montgomery-Asberg Depression Rating Scale; WHOQOL-BREF: Brief Version of World Health Quality of Life Inventory; FIQ: Fibromyalgia Impact Questionnaire; K-FIQ: Korean version of FIQ; BDI: Beck Depression Inventory; HADS: Hospital Anxiety and Depression Scale; VAS: Visual Analogue Scale; rTMS: repetitive Transcranial Magnetic Stimulation; BURS: Gracely Box Unpleasantness Rating Scales; MOS-SF-12: Medical Outcomes Study Short Form 12 items; GAF, Global Assessment of Functioning CGIS: Clinical Global Impression Scale; BAI: Beck Anxiety Inventory.

**Table 2:** Quality assessment scores (AMSTAR).

Study	1	2	3	4	5	6	7	8	9	10	11	12	13	Total
Choo et al. [17]	2	2	0	1	0	2	2	2	2	2	2	2	0	19
Knijnik et al. [16]	2	2	2	2	1	1	2	2	2	2	2	2	0	22
Hou et al. [15]	2	2	2	2	1	2	2	2	2	2	2	2	1	24
Saltychev & Laimi [18]	2	2	2	2	0	2	2	2	2	2	2	2	1	23
Conde-Antón et al. [19]	2	2	2	2	1	2	2	2	2	0	0	1	1	19
Kim et al. [20]	2	2	2	2	1	2	0	2	2	2	2	2	0	21
Toh et al. [21]	2	2	2	1	0	2	2	2	2	2	2	2	0	21
Sun et al. [22]	2	2	2	1	0	2	0	2	2	2	2	2	0	19
Su et al. [23]	2	2	2	2	0	2	0	2	2	2	2	1	0	19
Zhu et al. [24]	2	2	0	2	2	2	2	2	2	2	2	2	0	22
Marlow et al. [25]	1	2	2	2	0	1	2	0	2	0	0	1	0	13

1. Explicitly described to allow replication; 2. Adequate number and range of databases; 3. Alternative searches; 4. Adequate range of keywords; 5. Non-English-language papers included in the search; 6. Inclusion criteria explicitly described to allow replication; 7. Excludes reviews that do not adequately address inclusion and exclusion criteria; 8. Two independent reviewers assessed selection bias; 9. Quality assessment explicitly described to allow replication; 10. Meta-analysis conducted on only homogeneous data or limitations to homogeneity discussed; 11. Confidence intervals/effect sizes reported where possible; 12. Conclusions supported by the meta-analysis or other data analysis findings; 13. Conclusions address levels of evidence for each intervention/comparison.

**Table 3:** Risk of bias assessment in systematic reviews through ROBIS scale.

Study	Phase 2				Phase 3
	1. Study Eligibility Criteria	2. Identification and Selection of Studies	3. Data Collection and Study Appraisal	4. Synthesis and Findings	Risk of Bias in the Review
Choo et al. [17]	?	☺	☺	☹	☹
Knijnik et al. [16]	☺	☺	☺	☹	☺
Hou et al. [15]	☺	☺	☺	?	☺
Saltychev & Laimi [18]	☺	☺	☺	?	☺
Conde-Antón et al. [19]	☺	☺	☺	☹	☺
Kim et al. [20]	☺	☺	☺	☹	☺
Toh et al. [21]	☺	☺	☺	☹	☺
Sun et al. [22]	☺	☹	☺	☹	☹
Su et al. [23]	☺	☹	☺	☹	☹
Zhu et al. [24]	☺	☺	☺	☹	☺
Marlow et al. [25]	☹	☺	?	☹	☹

☺: low risk. ☹:= high risk. ?: unclear risk.

**Table 4:** Summary of findings and quality of evidence (PAGAC).

2018 Physical Activity Guidelines Advisory Committee Grading Criteria						Grade
Systematic Review Research Questions	Applicability	Generalizability	Risk of Bias or Study Limitations	Quantity and Consistency	Magnitude and Precision of Effect	
Pain Intensity	Strong	Limited	Limited	Limited	Not assignable	Limited
Depressive Symptoms	Strong	Limited	Moderate	Moderate	Not assignable	Moderate
Anxiety	Strong	Limited	Limited	Limited	Not assignable	Limited
General Health	Strong	Limited	Moderate	Moderate	Not assignable	Moderate

## Data Availability

Not applicable.

## Appendix A. Database Search Strategies

### Appendix A.1. Pubmed (Medline)

(“fibromyalgia”[MeSH Terms] OR “fibromyalgia”[All Fields] OR “fibromyalgias”[All Fields] OR (“fibromyalgia”[MeSH Terms] OR “fibromyalgia”[All Fields] OR (“fibromyalgia”[All Fields] AND “fibromyositis”[All Fields] AND “syndrome”[All Fields])) OR (“fibromyalgia”[MeSH Terms] OR “fibromyalgia”[All Fields] OR (“muscular”[All Fields] AND “rheumatism”[All Fields]) OR “muscular rheumatism”[All Fields]) OR (“fibromyalgia”[MeSH Terms] OR “fibromyalgia”[All Fields] OR “fibrositis”[All Fields]) OR (“fibromyalgia”[MeSH Terms] OR “fibromyalgia”[All Fields]) OR (“fibromyalgia”[MeSH Terms] OR “fibromyalgia”[All Fields] OR (“diffuse”[All Fields] AND “myofascial”[All Fields] AND “pain”[All Fields] AND “syndrome”[All Fields])) OR (“fibromyalgia”[MeSH Terms] OR “fibromyalgia”[All Fields] OR (“fibromyositis”[All Fields] AND “fibromyalgia”[All Fields] AND “syndrome”[All Fields])) OR (“fibromyalgia”[MeSH Terms] OR “fibromyalgia”[All Fields] OR (“fibromyalgia”[All Fields] AND “primary”[All Fields]) OR “fibromyalgia primary”[All Fields]) OR (“fibromyalgia”[MeSH Terms] OR “fibromyalgia”[All Fields] OR (“secondary”[All Fields] AND “fibromyalgia”[All Fields]) OR “secondary fibromyalgia”[All Fields])) AND (“transcranial magnetic stimulation”[MeSH Terms] OR (“transcranial”[All Fields] AND “magnetic”[All Fields] AND “stimulation”[All Fields]) OR “transcranial magnetic stimulation”[All Fields] OR (“symp theory model simul”[Journal] OR “tms”[All Fields]) OR (“transcranial magnetic stimulation”[MeSH Terms] OR (“transcranial”[All Fields] AND “magnetic”[All Fields] AND “stimulation”[All Fields]) OR “transcranial magnetic stimulation”[All Fields] OR “rtms”[All Fields]) OR “repetitive Transcranial Magnetic Stimulation”[All Fields] OR (“non-invasive”[All Fields] AND (“brain stimul”[Journal] OR (“brain”[All Fields] AND “stimulation”[All Fields]) OR “brain stimulation”[All Fields])) OR (“brain stimul”[Journal] OR (“brain”[All Fields] AND “stimulation”[All Fields]) OR “brain stimulation”[All Fields])) AND (“systematic review”[Publication Type] OR “systematic reviews as topic”[MeSH Terms] OR “systematic review”[All Fields] OR (“systematic reviews as topic”[MeSH Terms] OR (“systematic”[All Fields] AND “reviews”[All Fields] AND “topic”[All Fields]) OR “systematic reviews as topic”[All Fields]) OR (“meta analysis”[Publication Type] OR “meta analysis as topic”[MeSH Terms] OR “meta analysis”[All Fields]) OR (“meta analysis as topic”[MeSH Terms] OR (“meta analysis”[All Fields] AND “topic”[All Fields]) OR “meta analysis as topic”[All Fields] OR (“meta”[All Fields] AND “analysis”[All Fields] AND “topic”[All Fields]) OR “meta analysis as topic”[All Fields]) OR (“meta analysis”[Publication Type] OR “meta analysis as topic”[MeSH Terms] OR “meta analysis”[All Fields]))

### Appendix A.2. CINAHL (EBSCO)

(fibromyalgia or fibromyalgia syndrome or fms or fm) AND (transcranial magnetic stimulation or tms or rtms) AND (systematic review or meta-analysis)

- EMBASE

(‘fibromyalgia’/exp OR ‘fibromyalgia’ OR ‘fibrositic nodule’ OR ‘fibrositis’ OR ‘fibrositis syndrome’ OR ‘myalgia, fibro’) AND (‘transcranial magnetic stimulation’/exp OR ‘magnetic stimulation, transcranial’ OR ‘stimulation, transcranial magnetic’ OR ‘transcranial magnetic stimulation’ OR ‘repetitive transcranial magnetic stimulation’/exp OR ‘repetitive transcranial magnetic stimulation’ OR ‘transcranial magnetic stimulation, repetitive’) AND (‘systematic review’/exp OR ‘review, systematic’ OR ‘systematic review’ OR ‘meta analysis’/exp OR ‘analysis, meta’ OR ‘meta analysis’ OR ‘meta-analysis’ OR ‘meta-analysis’)

### Appendix A.3. PEDro

(a) Terms (Title & Abstract): Fibromyalgia AND rTMS

Methods: systematic review

(b) Terms (Title & Abstract): Fibromyalgia AND TMS

Methods: systematic review

(c) Terms (Title & Abstract): Fibromyalgia AND transcranial magnetic stimulation

Methods: systematic review

(d) Terms (Title & Abstract): Fibromyalgia AND repetitive transcranial magnetic stimulation

Methods: systematic review

(e) Terms (Title & Abstract): Fibromyalgia AND non-invasive brain stimulation

Methods: systematic review

(f) Terms (Title & Abstract): Fibromyalgia AND brain stimulation

Methods: systematic review

(g) Terms (Title & Abstract): Fibromyalgia AND electric stimulation

Methods: systematic review
